# Supplementary material for: Lactose Permease Scrambles Phospholipids
Source: Biology (Basel). 2023 Oct 25;12(11):1367. doi: 10.3390/biology12111367 (PMC10669175; doi:10.3390/biology12111367)
Supplement: Supplementary file 1 [file biology-12-01367-s001.zip › biology-2618473-supplementary.pdf]

Supplemental materials

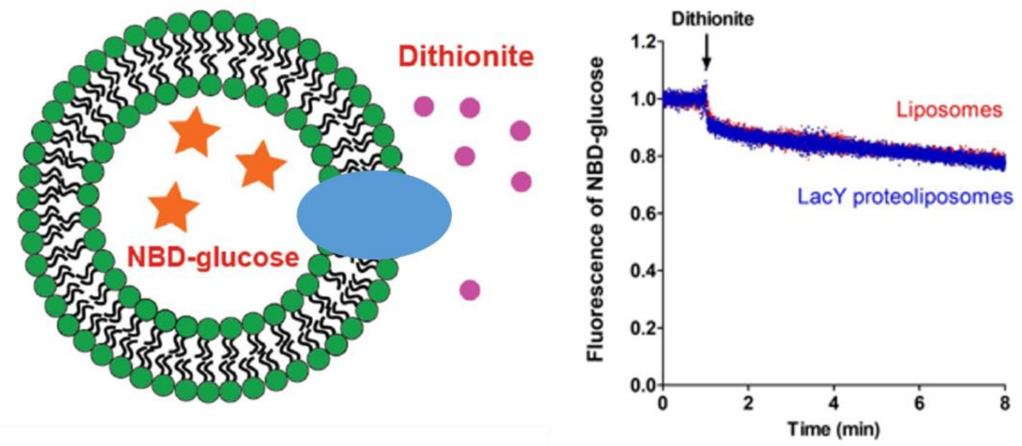

Figure S1: LacY does not affect dithionite permeability of liposomes. Liposomes were reconstituted in the presence of 50 μM NBD-glucose in the absence or presence of LacY, using a PPR of 2 mg/mmol. After removal of external NBD-glucose by centrifugation, dithionite was added after 1 min and fluorescence was recorded continuously for 8 min in mock-reconstituted liposomes (red trace) and LacY proteoliposomes (blue trace).

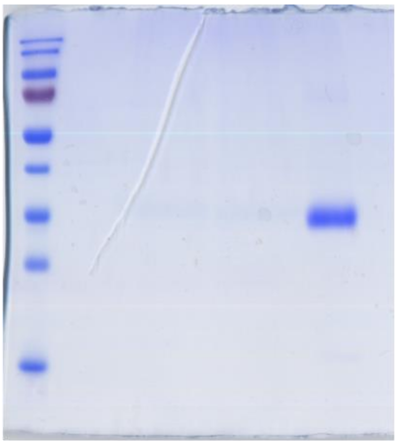

Fig. 1b

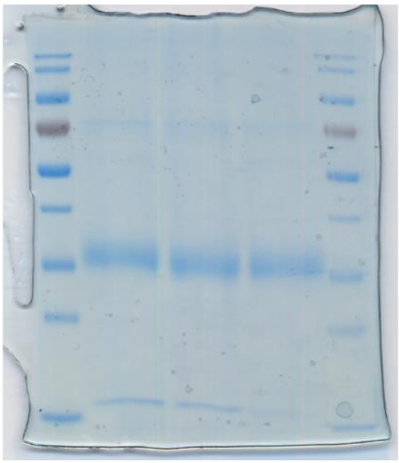

Fig. 3d

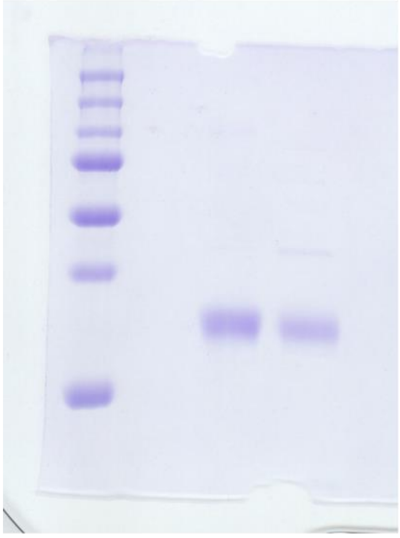

Fig. 4b and 5b

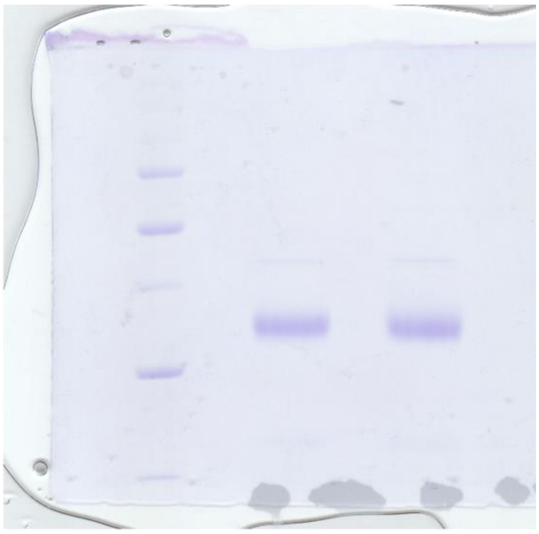

Fig. 6a and 6c

Figure S2: Original Coomassie-stained SDS-PAGE gels.
